# Mediation of depressive symptoms in the association between blood urea nitrogen to creatinine ratio and cognition among middle-aged and elderly adults: evidence from a national longitudinal cohort study

**DOI:** 10.1186/s12888-024-05941-7

**Published:** 2024-07-19

**Authors:** Qiaoduan Feng, Shaokun Yang, Shaohua Ye, Can Wan, Hongjian Wang, Jinsong You

**Affiliations:** 1 The Second Clinical College of Guangzhou, University of Chinese Medicine, Guangzhou, China; 2 West China School of Public Health and West China Fourth Hospital, Sichuan University, Chengdu, China; 3 Brain Cognition and Brain Disease Institute, Shenzhen Institute of Advanced Technology, Chinese Academy of Sciences, Shenzhen, China; 4 Department of Cerebrovascular Disease, The Second Affiliated Hospital of Guangzhou University of Chinese Medicine, Guangdong Provincial Hospital of Chinese Medicine, Guangzhou, China

**Keywords:** Blood urea nitrogen, Creatinine, Depressive symptoms, Cognition, Mediation

## Abstract

**Background:**

The relationships between BUNCr (blood urea nitrogen and creatinine ratio) and cognitive function, as well as depressive symptoms, remain unclear. We aim to investigate the association between BUNCr and cognition, as well as depressive symptoms, and to identify the mechanisms underlying these relationships.

**Methods:**

We utilized data from the China Health and Retirement Longitudinal Study (CHARLS) from 2015 to 2020. Cognitive function was assessed using the Telephone Interview of Cognitive Status (TICS) scale, while depressive symptoms were assessed using the 10-item Center for Epidemiologic Studies Depression Scale (CES-D-10). We employed multivariate linear regression models to examine the association between BUNCr and cognitive function, as well as depressive symptoms. Additionally, causal mediation analysis was conducted to identify potential mediation effects of depressive symptoms between BUNCr and cognition.

**Results:**

We observed a negative association between BUNCr and cognitive function (*coefficient*: -0.192; 95% confidence interval [*CI*]: -0.326 ∼ -0.059) and a positive relationship between BUNCr and depressive symptoms (*coefficient*: 0.145; 95% *CI*: 0.006 ∼ 0.285). In addition, the causal mediation analysis revealed that depressive symptoms (proportion mediated: 7.0%) significantly mediated the association between BUNCr and cognition.

**Conclusion:**

Our study has unveiled that BUNCr is inversely associated with cognitive function and positively linked to depressive symptoms. Moreover, we found that depressive symptoms significantly mediated the association between BUNCr and cognition. These findings provide new evidence and insights for the prevention and management of cognitive function and dementia.

**Supplementary Information:**

The online version contains supplementary material available at 10.1186/s12888-024-05941-7.

## Background

With the global population on the rise and the aging demographic becoming more pronounced, dementia has emerged as a pressing worldwide public health concern, as highlighted by the Global Burden of Disease (GBD) [1, 2]. In 2019, the GBD reported a staggering 57.4 million cases of dementia worldwide [3]. The decline in cognitive function represents a crucial transitional phase between normal cognitive status and full-blown dementia, potentially leading to irreversible neurodegenerative conditions and disabilities [4]. Moreover, when individuals lose their capacity for self-care and require assistance, this places a substantial economic burden on families and necessitates extensive medical resources [5]. Notably, there remains a shortage of effective drugs for the treatment of dementia [6]. In China, the prevalence of dementia has surged to 15.5% among those aged 60 or older in 2019, affecting approximately 38.77 million elderly individuals [7]. Since dementia cannot be entirely cured, it becomes imperative to prioritize the protection of cognitive function [8].

Depression is a prevalent mental disorder that has escalated into a global health crisis [9], ranking as a major contributor to the global disease burden with a worldwide prevalence of 5% [10]. Research suggests that depression is closely linked to various physical health conditions, including cardiovascular diseases, cerebrovascular diseases, malignancies, metabolic endocrine disorders, and infectious diseases [11]. The impact of depression on individuals extends to their quality of life and social engagement and can even lead to self-harm and suicidal tendencies [12, 13]. Furthermore, it places additional strain on family caregivers and escalates medical costs, leading to a significant economic burden [14].

Blood urea nitrogen (BUN) is a type of protein metabolite produced in the liver and excreted by the kidneys. It is often combined with creatinine (Cr) to create an important indicator known as the blood urea nitrogen and creatinine ratio (BUNCr), which is used to assess renal function in clinical practice [15]. Current studies have suggested that BUNCr is associated with various health outcomes and mortality. For example, Chen et al. found an association between BUNCr and physical frailty in a large population-based cohort of 5213 participants in China [16]. Nishioka et al. suggested that both higher and lower BUNCr levels may lead to poor neurological outcomes in an observational study conducted in Osaka, Japan [17]. Additionally, several studies have indicated that BUNCr is positively correlated with in-hospital mortality among patients with non-traumatic subarachnoid hemorrhage [18], acute myocardial infarction [19], and cardiogenic shock [20]. However, the relationships between BUNCr and cognitive function, as well as depression, are still unclear. Notably, a case-control study involving 75 participants revealed that elevated levels of creatinine and blood urea nitrogen may lead to white matter (WM) damage, which is essential for information processing across various brain regions [21]. This finding implies that BUNCr could potentially serve as a risk factor for cognitive impairment. Additionally, the role of depression in the association between BUNCr and cognition remains to be elucidated.

To address these gaps, we conducted the following analyses: (1) investigating the relationship between BUNCr and cognitive function; (2) exploring the association between BUNCr and depressive symptoms; (3) investigating the potential role of depressive symptoms in the connection between BUNCr and cognition. We aim for our study to offer robust evidence for the prevention and management of cognitive decline and dementia.

## Methods

### Study design and participants

The individuals included in our analysis, who participated in all waves 3, 4, and 5, were selected from the China Health and Retirement Longitudinal Study (CHARLS). CHARLS, a longitudinal cohort study, has collected data across five waves in the years 2011, 2013, 2015, 2018, and 2020. Using a stratified, multistage probability sampling method, the baseline survey (Wave 1) involved 17,708 individuals aged 45 or older, representing middle-aged and elderly populations on a national scale in China across 150 districts and counties and 450 villages. The data encompasses diverse information obtained through face-to-face interviews, physical examinations, and blood biochemical tests, covering demographic characteristics, lifestyle and behavioral traits, physical and psychological health, as well as biomarkers, among other factors. The CHARLS study received approval from the Peking University Biomedical Ethics Committee (IRB00001052-11015), and all participants provided informed consent. Further detailed information about this study can be found elsewhere [22].

**Fig. 1:**
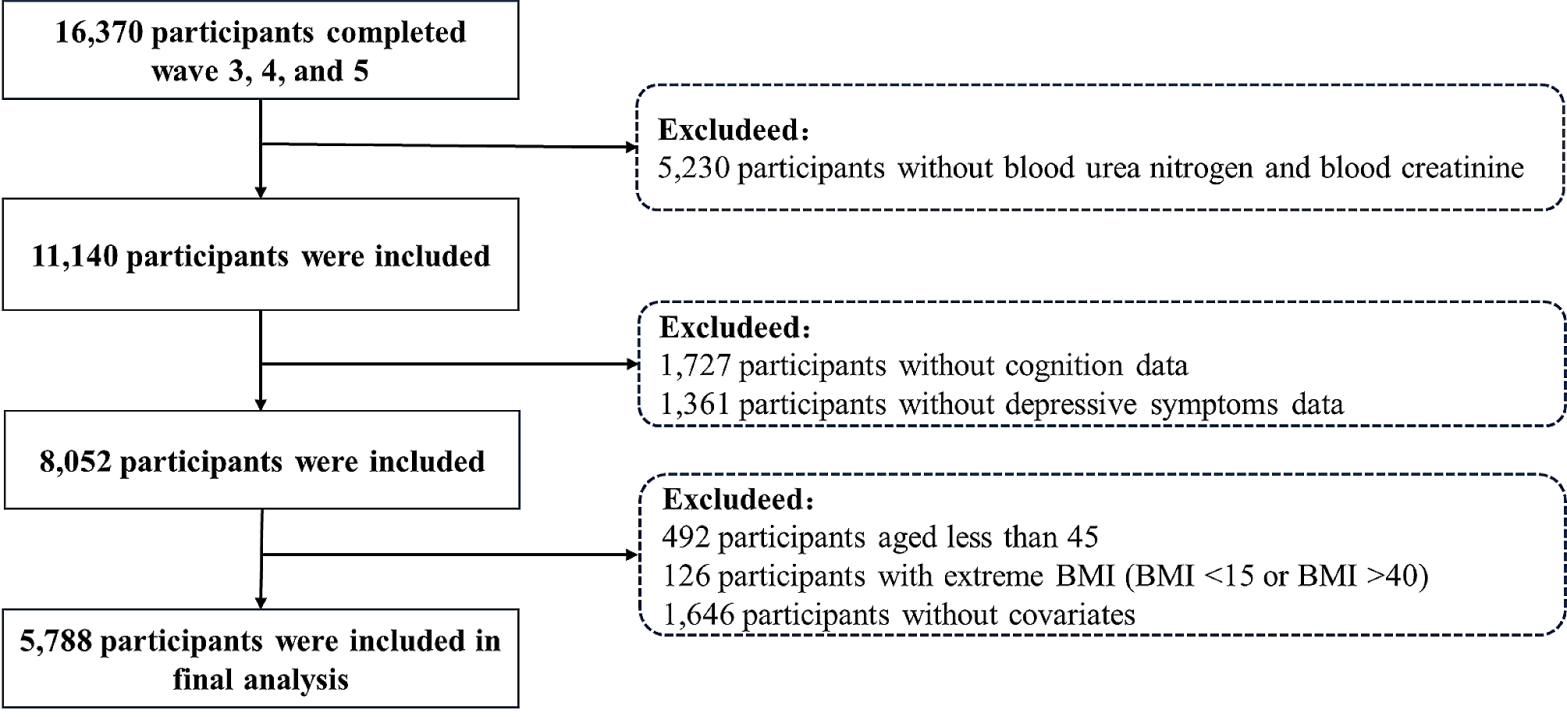
Flow chart of participants included

16,370 participants were involved in all waves 3, 4, and 5, but only 11,140 had data on blood urea nitrogen and blood creatinine. After excluding 1,727 participants without cognition and 1,361 participants without depressive symptoms, 8,052 participants remained. Additionally, individuals who: (1) were under 45 years old; (2) had an extreme BMI; and (3) lacked covariates were also excluded. Ultimately, 5,788 individuals meeting our criteria were included in the final analysis. The process is outlined in Fig. 1.

### Assessment of cognition

Cognitive function in CHARLS was assessed using the Telephone Interview of Cognitive Status (TICS) scale [23, 24], which was adapted from the Health and Retirement Study (HRS) in the USA. The TICS test consists of two primary components: episodic memory and mental status, reflecting both fluid and crystallized intelligence. These components encompass orientation, visuoconstruction, and numeric ability. Episodic memory was measured through immediate and delayed recall. Respondents were required to promptly recall ten Chinese words presented by the investigator and subsequently recollect the same ten Chinese words after a brief delay. The score for this segment was based on the number of words correctly recalled, with scores ranging from 0 to 20. Orientation was assessed through five items related to the current day, week, month, season, and year. The respondent’s score depended on the number of items correctly answered, ranging from 0 to 5. Visuoconstruction was evaluated by instructing the respondent to accurately draw a picture shown by the investigator. Numeric ability was assessed by asking respondents to answer a mathematical question: “What is the result of subtracting 7 once, twice, three times, four times, and five times from 100?” The score for this part was determined by the number of correct answers, ranging from 0 to 5. Mental status was represented by the sum of the points from the orientation, visuoconstruction, and numeric ability sections, resulting in a score ranging from 0 to 11. In the end, the total score from these two main components ranged from 0 to 31, with higher scores indicating better cognitive function.

### Measurement of blood urea nitrogen and creatinine

After conducting face-to-face interviews in districts or counties, respondents were asked to visit local hospitals or the Center for Disease Prevention and Control (CDC), where trained nurses collected 8-ml blood samples. The blood sample analysis involved two steps. Initially, complete blood count (CBC) tests, including hemoglobin, hematocrit, white blood cell count (WBC), platelet count, and mean corpuscular volume (MCV), were performed within 2 h at the local hospital or CDC using 2-ml blood samples. Subsequently, the remaining blood samples were stored at -20 °C in cryovials and transported to the study headquarters in Beijing for further analysis. These samples were utilized to assay for blood urea nitrogen (mg/dL), creatinine (mg/L), and other blood biomarkers. Further detailed information about blood samples can be accessed elsewhere [25]. BUNCr was calculated by dividing blood urea nitrogen by creatinine. Both measurements have units of ml/dL, so the ratio has a unit of one. BUNCr divided by the standard deviation was used in the following statistical analyses.

### Assessment of depressive symptoms

The 10-item Center for Epidemiologic Studies Depression Scale (CES-D-10) was utilized to assess depressive symptoms. This scale has been validated as an effective screening tool for depressive symptoms among middle-aged and older Chinese adults [26]. Each item measured the frequency of a specific negative mood, with response options ranging from 0 for “rarely or none” to 3 for “most of the time.” The total score, ranging from 0 to 30, was used to evaluate the overall status of depressive symptoms, with higher scores indicating a higher level of depressive symptoms. To categorize the CES-D-10 score and assess depressive symptoms status, a cut-off value of 12 was employed [27].

### Covariates

Consistent with prior research [28, 29], we examined a range of demographic characteristics, behavioral traits, and health conditions. Demographic characteristics included gender (female and male), age, marital status (living with a partner and living without a partner), educational level (ranging from illiteracy to elementary, secondary, and higher education), and residence (urban and rural). Behavioral traits encompassed alcohol consumption (drink or quit, and never) and smoking (smoke or quit, and never). Health conditions were evaluated based on body mass index (BMI) and the presence of hypertension, diabetes, and dyslipidemia (yes or no).

### Statistical analysis

The BUNCr, depressive symptoms, and cognitive function used in our analysis were assessed in 2015 (wave 3), wave 2018 (wave 4), and wave 2020 (wave 5), respectively. BUNCr were categorized into four groups (Q1, Q2, Q3, and Q4) based on their 25th percentile, median, and 75th percentile values. Quantitative variables were summarized using the mean ± standard error (SD), while qualitative variables were presented as counts (%) within each group. Statistical analysis involved one-way ANOVA and chi-square tests to compare variables among the four groups.

We utilized a multiple linear regression model to explore the association between BUNCr and cognitive function as well as depressive symptoms. Model 1 was conducted without the inclusion of any covariates. In Model 2, we integrated gender, age, marital status, education, and residence as covariates. Model 3 involved additional adjustments for alcohol and smoking. Furthermore, Model 4 accounted for hypertension, diabetes, and dyslipidemia conditions. The findings were reported in the form of coefficients and corresponding confidence intervals (*CI*).

We utilized Restricted Cubic Spline (RCS) linear regression with three knots (10th, 50th, and 90th quantiles) to evaluate the non-linear association between BUNCr and both cognitive function and depressive symptoms. The model was adjusted for the same set of covariates as in Model 4.

In order to investigate the mechanism of BUNCr on cognitive function, we employed causal mediation analysis, guided by four key assumptions [30]: (1) the absence of unmeasured confounding factors between the exposure and outcome; (2) the absence of unmeasured confounding factors between the exposure and mediator; (3) the absence of unmeasured confounding factors between the mediator and outcome; (4) the confounding factors between the exposure and mediator were not influenced by the exposure. The total effect (TE), natural direct effect (NDE), and natural indirect effect (NIE) estimated by the natural effect model proposed by VanderWeele [31] would hold causal significance, provided that the aforementioned four assumptions were met. We obtained the proportion mediated (PM) by dividing the NIE by TE. Based on the general approach to causal mediation analysis proposed by Imai [32], we conducted four mediation analyses (M1-4). M1 was not adjusted for any covariates. M2 was adjusted for sex, age, marriage, education, and residence. M3 was additionally adjusted for alcohol and smoking. M4 additionally took into account hypertension, diabetes, and dyslipidemia conditions. The causal mediation analysis was executed using the R package “mediation”, which estimates the confidence intervals for TE, NDE, NIE, and PM via bootstrapping with 5000 resamples [33].

We conducted a series of sensitivity analyses to confirm the reliability of our results. Stratified analysis was performed to identify potential effect modifiers for the relationship between BUNCr and both cognitive function and depressive symptoms. In each scenario, we carried out multiple regression, causal mediation analysis, and stratified analysis separately. These scenarios included: (1) Employing a multiple imputation approach to handle missing data for covariates. (2) Including participants under 45 years old and those with extreme BMI values. (3) Using episodic memory and mental status as outcomes. Furthermore, we calculated the E-value to quantify the strength of association that an unmeasured confounder would need to have with both the exposure and the outcome in order to fully explain away the observed effect [34].

All the statistical analyses were performed using R software (version 4.3.1), which is available at https://www.r-project.org. The *P* value of both-sides test less than 0.05 was supposed to be statistically significant.

## Results

### Baseline characteristics

In our final analysis, a total of 5,788 participants were included, with an average age of 59.26 ± 7.97. The population demonstrated an average cognitive score of 16.87 ± 5.59 and an average depressive symptoms score of 9.77 ± 5.38. Among the participants, 3,007 (52.0%) were female, and 2,781 (48.0%) were male. We divided the population into four groups based on BUNCr quantiles. The average cognitive scores for the four groups were 17.34 ± 5.53, 17.15 ± 5.42, 16.87 ± 5.65, and 16.13 ± 5.69, indicating a decreasing trend from Q1 to Q4. In contrast, the average depressive symptoms scores for the four groups were 9.13 ± 5.11, 9.73 ± 5.14, 9.84 ± 5.53, and 10.37 ± 5.66, showing an increasing trend from Q1 to Q4. Further details regarding the basic characteristics of the population are provided in Table 1.

**Table 1:** Basic characteristics of the population [*n* (%) or Mean ± SD]

Characteristic	Overall, *N* = 5,786	Q1 *N* = 1,447	Q2 *N* = 1,447	Q3 *N* = 1,445	Q4 *N* = 1,447	*P* ^a^
**Cognition**	16.87 ± 5.59	17.34 ± 5.53	17.15 ± 5.42	16.87 ± 5.65	16.13 ± 5.69	< 0.001
**Depressive symptoms**	9.77 ± 5.38	9.13 ± 5.11	9.73 ± 5.14	9.84 ± 5.53	10.37 ± 5.66	< 0.001
**Gender**						< 0.001
Femal	3,007 (52.0)	498 (34.4)	677 (46.8)	837 (57.9)	995 (68.8)	
Male	2,781 (48.0)	949 (65.6)	771 (53.2)	609 (42.1)	452 (31.2)	
**Age**	59.26 ± 7.97	59.37 ± 8.38	58.95 ± 7.90	59.35 ± 7.87	59.37 ± 7.70	0.418
**Marriage**						0.267
With partner	4,992 (86.2)	1,235 (85.3)	1,237 (85.4)	1,265 (87.5)	1,255 (86.7)	
Without partner	796 (13.8)	212 (14.7)	211 (14.6)	181 (12.5)	192 (13.3)	
**Education**						< 0.001
Elementary	3,516 (60.7)	799 (55.2)	835 (57.7)	897 (62.0)	985 (68.1)	
Secondary	2,181 (37.7)	615 (42.5)	583 (40.3)	533 (36.9)	450 (31.1)	
Higher	91 (1.6)	33 (2.3)	30 (2.1)	16 (1.1)	12 (0.8)	
**Residence**						< 0.001
Urban	1,168 (20.2)	361 (24.9)	311 (21.5)	282 (19.5)	214 (14.8)	
Rural	4,620 (79.8)	1,086 (75.1)	1,137 (78.5)	1,164 (80.5)	1,233 (85.2)	
**Alcohol**						< 0.001
No	3,035 (52.4)	625 (43.2)	739 (51.0)	800 (55.3)	871 (60.2)	
drink or quit	2,753 (47.6)	822 (56.8)	709 (49.0)	646 (44.7)	576 (39.8)	
**Smoke**						< 0.001
No	3,235 (55.9)	628 (43.4)	765 (52.8)	881 (60.9)	961 (66.4)	
smoke or quit	2,553 (44.1)	819 (56.6)	683 (47.2)	565 (39.1)	486 (33.6)	
**BMI**	24.13 ± 3.63	23.93 ± 3.63	24.17 ± 3.50	24.25 ± 3.54	24.16 ± 3.83	0.092
**Hypertension**						0.457
No	3,961 (68.4)	1,013 (70.0)	990 (68.4)	984 (68.0)	974 (67.3)	
Yes	1,827 (31.6)	434 (30.0)	458 (31.6)	462 (32.0)	473 (32.7)	
**Diabetes**						0.039
No	5,215 (90.1)	1,326 (91.6)	1,299 (89.7)	1,309 (90.5)	1,281 (88.5)	
Yes	573 (9.9)	121 (8.4)	149 (10.3)	137 (9.5)	166 (11.5)	
**Dyslipidemia**						0.370
No	4,646 (80.3)	1,155 (79.8)	1,162 (80.2)	1,146 (79.3)	1,183 (81.8)	
Yes	1,142 (19.7)	292 (20.2)	286 (19.8)	300 (20.7)	264 (18.2)	

^a^*P* value was caculated for continuous variables by t-test and categorical variables by Pearson’s Chi-squared test

Abbreviations: SD, stanard deviation; BMI, body mass index

### The relationships between BUNCr and both cognition and depressive symptoms

**Fig. 2:**
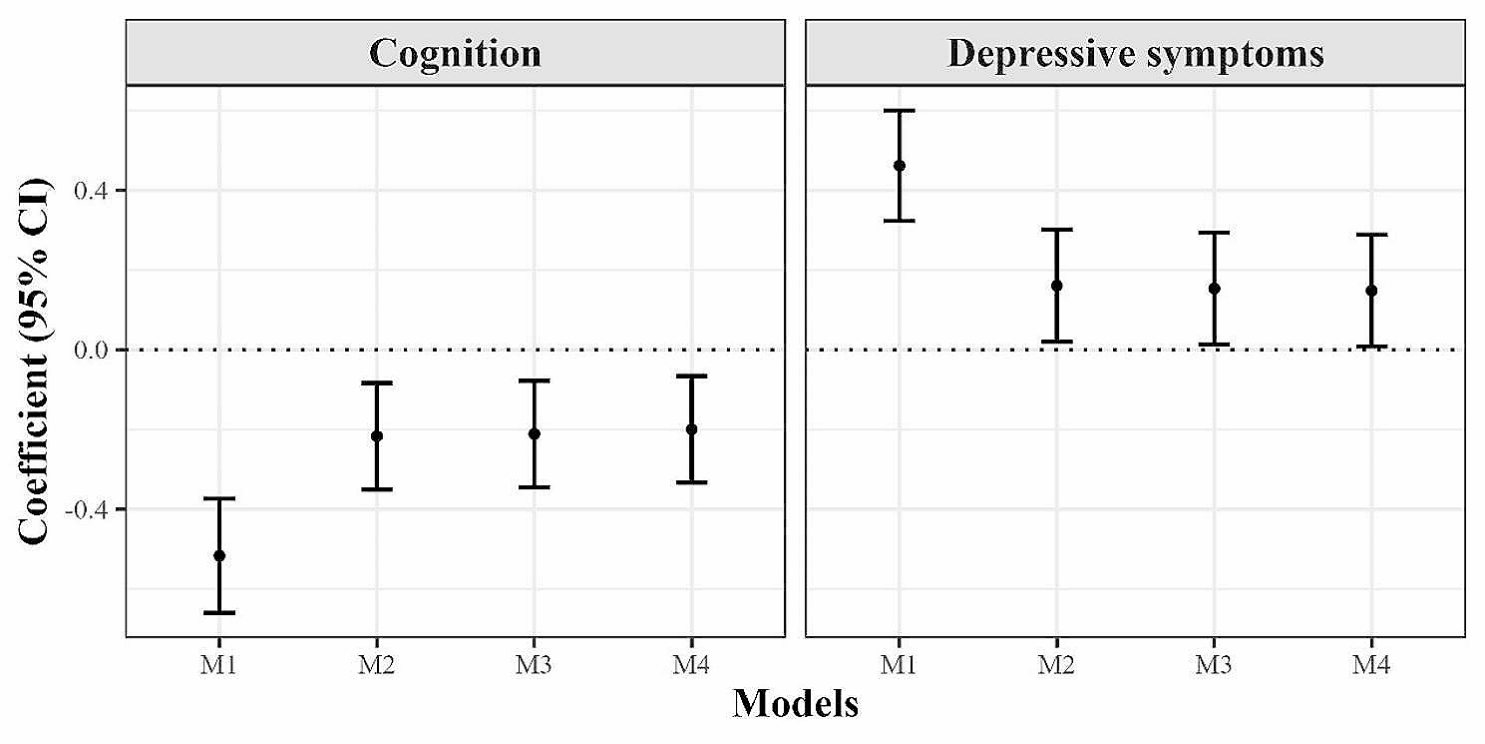
The relationships between BUNCr and both cognitive function and depressive symptoms. Abbreviations: BUNCr, blood urea nitrogen to creatinine ratio. CI, confidence interval. Model 1 adjusted for no covariates. Model 2 adjusted for age, marital status, education, and residence. Model 3 adjusted for age, marital status, education, residence, alcohol, and smoking. Model 4 adjusted for age, marital status, education, residence, alcohol, and smoking, BMI, hypertension, diabetes, and dyslipidemia

We conducted four models for cognition, all of which consistently demonstrated a negative association between BUNCr and cognitive function. Similarly, in the case of depressive symptoms, the four models consistently indicated a positive relationship between BUNCr and depressive symptoms (refer to Fig. 2). For example, in the fully adjusted model 4 for cognition, the coefficient (95% *CI*) was − 0.192 (-0.326,-0.059), and in the fully adjusted model 4 for depressive symptoms, the coefficient (95% *CI*) was 0.145 (0.006,0.285). The coefficients for cognition and depressive symptoms remained stable across models 2 to 4. Detailed numerical results corresponding to Fig. 2 are provided in Table S1. We also found depressive symptoms and BUN were negatively associated with cognitive function too. (Table S2-3).

### The concentration-reponse curve between BUNCr and both cognitive function and depressive symptoms

**Fig. 3:**
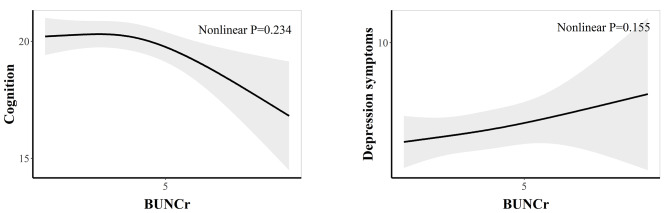
The concentration-reponse curve between BUNCr and both cognitive function and depressive symptoms. Abbreviations: BUNCr, blood urea nitrogen to creatinine ratio. Model adjusted for age, marital status, education, residence, alcohol, and smoking, BMI, hypertension, diabetes, and dyslipidemia

In addition, we conducted RCS analysis to explore the linear relationship between BUNCr and both cognitive function and depressive symptoms. The findings indicated that the relationships were predominantly linear, as depicted in Fig. 3. The p-values for the non-linear tests were 0.233 and 0.155 for cognition and depressive symptoms, respectively.

### Causal mediation analysis

**Fig. 4:**
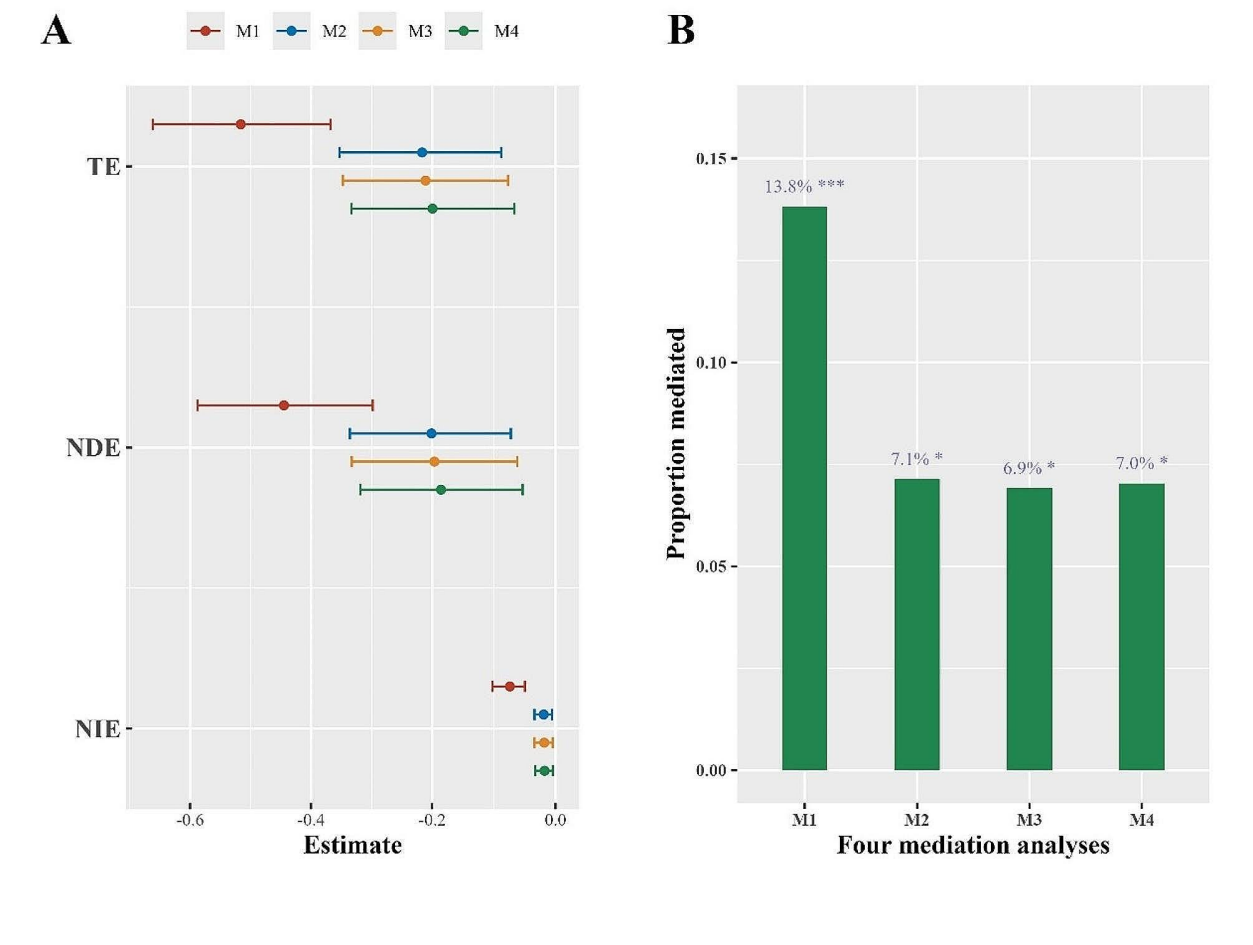
Mediation analyses of depressive symptoms on BUNCr-induced cognition. **A**: The TE, NDE, and NIE of BUNCr on cognition; **B**: The proportion mediated by depressive symptoms on the relationship between BUNCr and cognition. Abbreviations: BUNCr, blood urea nitrogen to creatinine ratio. CI, confidence interval. M1 adjusted for no covariates. M2 adjusted for age, marital status, education, and residence. M3 adjusted for age, marital status, education, residence, alcohol, and smoking. M4 adjusted for age, marital status, education, residence, alcohol, and smoking, BMI, hypertension, diabetes, and dyslipidemia. *,*P* < 0.05; **,*P* < 0.01; ***,*P* < 0.001

In the fully adjusted model, the total effect (95% *CI*), direct effect (95% *CI*), and indirect effect (95% *CI*) were − 0.1992 (-0.3331,-0.0638), -0.1852 (-0.3186,-0.0502), and − 0.014 (-0.0293,-0.0005), respectively (Fig. 4). The proportion mediated by depressive symptoms between BUNCr and cognition was 7% (Fig. 4). Detailed numerical results corresponding to Fig. 4 are provided in Table S4. It is noteworthy that, after adjusting for covariates, the TE, NDE, NIE, and PE remained similar across M2 to M4.

### Sensitivity analysis

Stratified analysis demonstrated that smoking modified the relationship between BUNCr and cognition. Non-smokers were more susceptible compared with those who were currently smoking or quit (*P*_int_ = 0.031). After employing multiple imputation for missing covariates, as well as the inclusion of participants under 45 years old and those with extreme BMI values, and using episodic memory and mental status as outcomes, the results from the multiple linear regression (Table S7-10), causal mediation analysis (Table S11-15), and stratified analyses (Table S16-23) remained robust. The E-values for cognition and depressive symptoms were 1.21 and 1.18, indicating that the impact of unmeasured confounding on the results may not be substantial.

## Discussion

Based on a large nationwide cohort study that included over twenty thousand middle-aged and elderly participants in China, our research revealed a negative association between BUNCr and cognitive function, as well as a positive relationship between BUNCr and depressive symptoms. Furthermore, depressive symptoms were found to significantly mediate the association between BUNCr and cognitive function. This study is believed to be the first and largest epidemiological investigation to explore the relationship between BUNCr and both cognitive function and depressive symptoms.

### Comparison with other studies

Our research first found that BUNCr was negatively associated with cognitive function and positively related to depressive symptoms. To date, the association between BUNCr and cognition function as well as depressive symptoms has not been extensively studied.

On the one hand, for cognition, our research has some similarities with many previous studies. Existing studies have shown that elevated BUNCr increases the risk of cognitive impairment. For example, a retrospective cohort study of 7167 patients admitted to the intensive care unit found that BUNCr was associated with hypoactive delirium (OR = 1.012, *P* < 0.05) [35]. Another prospective cohort study of 135 subjects in the United States showed that BUNCr was an independent risk factor for delirium (RR = 2.9, *P* < 0.05) [36]. In addition, delirium, an acute cognitive and attentional disorder [37], is inextricably linked to cognitive function. A meta-analysis of 23 studies showed that delirium was associated with long-term cognitive decline, and the combined OR (95% *CI*) was 2.30 (1.85–2.86) [38]. These studies suggest that BUNCr may contribute to cognitive impairment, which in turn may lead to cognitive decline. This is consistent with our results, but the role of delirium in the relationship between BUNCr and cognitive decline warrants further exploration.

On the other hand, in terms of depressive symptoms, to the best of our knowledge, our study is the first epidemiologic study to explore the relationship between BUNCr and depressive symptoms in a middle-aged or older population. Previous studies have explored the relationship between blood urea nitrogen or blood creatinine and depression or depressive symptoms, respectively. For example, a cross-sectional study from Jordan found that blood urea nitrogen and creatinine could serve as risk predictors for depressive symptoms. Therefore, more epidemiologic studies from different countries, regions, and populations, as well as experimental animal studies, are needed to further explore this.

### Potential mechanisms

The mechanisms of the relationships between BUNCr and cognitive function as well as depressive symptoms remain largely unclear, but some animal research and population-based studies can provide us with some clues.

For cognition, firstly, BUN may change the microstructures of the brain and cause the brain to shrink. In a study aimed at examining the association between brain microstructure and physiological indicators, axial diffusivity, radial diffusivity, and mean diffusivity values that indicate changes in the microstructure of the brain white matter were found to be significantly positively associated with BUN [39]. And another survey found that urea nitrogen levels were correlated with cortical thickness and volume among patients with CKD, and patients with CKD potentially exhibit a certain degree of structural brain-tissue imaging changes, with morphological changes more pronounced in patients with DD-CKD, suggesting that blood urea nitrogen and dialysis may be influential factors in brain morphological changes in patients with CKD [40]. Secondly, BUN is associated with abnormal activity in the brain. Low-frequency fluctuations showed a significantly positive relationship with urea nitrogen among patients with CKD, which means that abnormal activity in the basal ganglia, cerebellum, and hippocampal regions may be involved in cognitive decline in CKD patients [41]. Thirdly, BUN was found to be negatively associated with the diffusion tensor imaging analysis along the perivascular space (DTI-ALPS) index, which indicates perivascular glymphatic activity. While lower DTI-ALPS was related to higher glial fibrillary acidic protein, an indicator for astrocyte injury, astrocyte impairment may affect the transportation of astrocytic aquaporin-4 in the perivascular space, causing a decrease in cognitive function [42]. Finally, BUNCr is a biomarker of kidney function. We speculate that it is related to renal insufficiency, which in turn leads to a decline in cognitive function [43].

For depressive symptoms, firstly, findings from an animal experiment revealed that blood urea can disrupt long-term potentiation and lead to significant loss of synapses, ultimately inducing symptoms of depression [44]. Secondly, it was observed that blood urea contributes to the development of depressive symptoms by impairing the medial prefrontal cortex [45]. Thirdly, the urea transporter protein UT-B was found to play a critical role in urea transport within the brain, particularly in the hippocampus. The absence of UT-B results in urea accumulation in the hippocampus, potentially triggering depression-like behavior by interfering with the nitric oxide synthase/nitric oxide (NO_S_/NO) system [46].

Our stratified analysis revealed that non-smokers exhibited greater susceptibility compared to current or former smokers, which contrasts with previous findings suggesting that smoking may lead to cognitive decline [47, 48]. This disparity could be attributed to several factors. Firstly, individuals who smoke may have more social interactions and receive increased social support, potentially counteracting the negative effects of BUNCr. Additionally, prior research has indicated that nicotine can enhance cognitive function [49, 50].

However, the underlying mechanisms behind these findings still require further investigation.

### Mediation analysis

The causal mediation analysis indicated the pathway “BUNCr-depressive symptoms-cognition”. Our findings demonstrate robustness as they remain consistent across different models adjusting for various covariates in the primary analysis (Models 2–4) as well as in the sensitivity analysis involving diverse population subsets. This consistency reinforces the reliability of our results, supporting the validity of the observed associations.

The proportion mediated by depressive symptoms on the association between BUNCr and cognition was 7%, suggesting that intervening on depressive symptoms could potentially reduce the impact of BUNCr on cognition. This is particularly important as the decline in cognitive function may lead to irreversible dementia.

### Strengths and limitations

Our research has several strengths. Firstly, we utilized a large national population cohort, enabling us to gather a comprehensive set of variables, including demographic characteristics, behavioral patterns, health status, as well as physiological and biochemical indicators. This extensive data collection ensures the reliability and accuracy of our study. Secondly, BUNCr, depressive symptoms, and cognition were collected in 2015, 2018, and 2020, respectively, avoiding causal inversion as much as possible. Thirdly, our research represents the first to identify a correlation between BUNCr and cognitive function, as well as depressive symptoms, and to uncover the mediating role of depressive symptoms. Lastly, we conducted multiple sensitivity analyses to enhance the robustness of our results.

However, our research does have certain limitations. Firstly, the reliance on participants’ recollection for some variables raises the potential for recall bias. Secondly, despite our diligent efforts to control for confounding variables, there is still the possibility of unmeasured confounding leading to bias. Thirdly, while we assessed cognitive function across multiple domains, our evaluation may still be incomplete, and there may be other relevant aspects that were not fully captured. Finally, our biochemical indicators were measured only once. Obtaining multiple measurements and analyzing the average result may enhance the precision and reliability of our findings.

## Conclusions

In summary, our study is the first to establish a link between BUNCr and cognitive function as well as depressive symptoms in a large-scale cohort of middle-aged and elderly Chinese individuals. Our findings indicate that elevated BUNCr is associated with a decline in cognitive function and an exacerbation of depressive symptoms, with depressive symptoms mediating the relationship between BUNCr and cognition. This research provides valuable evidence for improving cognitive function, alleviating depressive symptoms, and developing new methods for protecting against dementia and depressive symptoms.

### Electronic supplementary material

Below is the link to the electronic supplementary material.

Supplementary Material 1

## Data Availability

The data used in this paper is available in public and can be accessed at China Healthand Retirement Longitudinal Study (CHARLS) https://charls.pku.edu.cn/.

## Abbreviations

GBD: Global Burden of Disease
BUN: Blood urea nitrogen
Cr: Creatinine
BUNCr: Blood urea nitrogen and creatinine ratio
CHARLS: China Health and Retirement Longitudinal Study
TICS: Telephone Interview of Cognitive Status
HRS: Health and Retirement Study
CDC: Center for Disease Prevention and Control
WBC: White blood cell count
CES-D-10: 10-item Center for Epidemiologic Studies Depression scale
BMI: Body mass index
SD: Standard error
CI: Confidence intervals
RCS: Restricted Cubic Spline
TE: Total effect
NDE: Natural direct effect
NIE: Natural indirect effect
AHF: Acute heart failure
CKD: Chronic kidney disease
