# Supplementary material for: Mediation of depressive symptoms in the association between blood urea nitrogen to creatinine ratio and cognition among middle-aged and elderly adults: evidence from a national longitudinal cohort study
Source: BMC Psychiatry. 2024 Jul 19;24:515. doi: 10.1186/s12888-024-05941-7 (PMC11264492; doi:10.1186/s12888-024-05941-7)
Supplement: Supplementary file 1 — Supplementary Material 1 [file 12888_2024_5941_MOESM1_ESM.docx]

**Supplemental Materials**

**Contents**

[Table S1 The relationships between BUNCr and both cognitive function and depressive symptoms 2](#_Toc153803134)

[Table S2 The relationships between cognitive function and depressive symptoms 3](#_Toc153803135)

[Table S3 The relationships between BUN and both cognitive function and depressive symptoms 4](#_Toc153803136)

[Table S4 Mediation analyses of depressive symptoms on BUNCr-induced cognition 5](#_Toc153803137)

[Table S5 The relationships between BUNCr and cognitive function stratified by potential modifiers 6](#_Toc153803138)

[Table S6 The relationships between BUNCr and depressive symptoms stratified by potential modifiers 7](#_Toc153803139)

[Table S7 The relationships between BUNCr and both cognitive function and depressive symptoms after multiple imputation 8](#_Toc153803140)

[Table S8 The relationships between BUNCr and both cognitive function and depressive symptoms after including participants under 45 years old 9](#_Toc153803141)

[Table S9 The relationships between BUNCr and both cognitive function and depressive symptoms after including participants with extreme BMI 10](#_Toc153803142)

[Table S10 The relationships between BUNCr and both episodic memory and mental status 11](#_Toc153803143)

[Table S11 Mediation analyses of depressive symptoms on BUNCr-induced cognition after multiple imputation 12](#_Toc153803144)

[Table S12 Mediation analyses of depressive symptoms on BUNCr-induced cognition after including participants under 45 years old 13](#_Toc153803145)

[Table S13 Mediation analyses of depressive symptoms on BUNCr-induced cognition after including participants with extreme BMI 14](#_Toc153803146)

[Table S14 Mediation analyses of depressive symptoms on the relationship between BUNCr and episodic memory 15](#_Toc153803147)

[Table S15 Mediation analyses of depressive symptoms on the relationship between BUNCr and mental status 16](#_Toc153803148)

[Table S16 The relationships between BUNCr and cognitive function stratified by potential modifiers after multiple imputation 17](#_Toc153803149)

[Table S17 The relationships between BUNCr and depressive symptoms stratified by potential modifiers after multiple imputation 18](#_Toc153803150)

[Table S18 The relationships between BUNCr and cognitive function stratified by potential modifiers after including participants with extreme BMI 19](#_Toc153803151)

[Table S19 The relationships between BUNCr and depressive symptoms stratified by potential modifiers after including participants under 45 years old 20](#_Toc153803152)

[Table S20 The relationships between BUNCr and cognitive function stratified by potential modifiers after including participants with extreme BMI 21](#_Toc153803153)

[Table S21 The relationships between BUNCr and depressive symptoms stratified by potential modifiers after including participants with extreme BMI 22](#_Toc153803154)

[Table S22 The relationships between BUNCr and episodic memory stratified by potential modifiers 23](#_Toc153803155)

[Table S23 The relationships between BUNCr and mental status stratified by potential modifiers 24](#_Toc153803156)

**Table S1 The relationships between BUNCr and both cognitive function and depressive symptoms**

| **Outcome** | **Model** | **Coefficient (95% *CI*)** | ***P*** |
| --- | --- | --- | --- |
| Cognition | Model 1 | -0.516(-0.660,-0.373) | <0.001 |
| Cognition | Model 2 | -0.216(-0.350,-0.083) | 0.001 |
| Cognition | Model 3 | -0.211(-0.344,-0.077) | 0.002 |
| Cognition | Model 4 | -0.192(-0.326,-0.059) | 0.005 |
| Depressive symptoms | Model 1 | 0.462(0.323,0.600) | <0.001 |
| Depressive symptoms | Model 2 | 0.161(0.021,0.301) | 0.025 |
| Depressive symptoms | Model 3 | 0.154(0.014,0.294) | 0.032 |
| Depressive symptoms | Model 4 | 0.145(0.006,0.285) | 0.042 |

Abbreviations: BUNCr, blood urea nitrogen to creatinine ratio; CI, confidence interval.

Model 1 adjusted for no covariates.

Model 2 adjusted for age, gender, marital status, education, and residence.

Model 3 adjusted for age, gender, marital status, education, residence, alcohol, and smoking.

Model 4 adjusted for age, gender marital status, education, residence, alcohol, and smoking, BMI, hypertension, diabetes, and dyslipidemia.

**Table S2 The relationships between cognitive function and depressive symptoms**

| **Model** | **Coefficient (95% *CI*)** | ***P*** |
| --- | --- | --- |
| Model 1 | -0.162(-0.188,-0.135) | <0.001 |
| Model 2 | -0.097(-0.122,-0.073) | <0.001 |
| Model 3 | -0.096(-0.120,-0.071) | <0.001 |
| Model 4 | -0.094(-0.118,-0.070) | <0.001 |

Abbreviations: CI, confidence interval.

Model 1 adjusted for no covariates.

Model 2 adjusted for age, gender, marital status, education, and residence.

Model 3 adjusted for age, gender, marital status, education, residence, alcohol, and smoking.

Model 4 adjusted for age, gender marital status, education, residence, alcohol, and smoking, BMI, hypertension, diabetes, and dyslipidemia.

**Table S3 The relationships between BUN and both cognitive function and depressive symptoms**

| **Outcome** | **Model** | **Coefficient (95% *CI*)** | ***P*** |
| --- | --- | --- | --- |
| Cognition | Model 1 | -0.068(-0.102,-0.035) | <0.001 |
| Cognition | Model 2 | -0.052(-0.083,-0.021) | 0.001 |
| Cognition | Model 3 | -0.052(-0.082,-0.021) | 0.001 |
| Cognition | Model 4 | -0.048(-0.078,-0.017) | 0.002 |
| Depressive symptoms | Model 1 | -0.021(-0.054,0.011) | 0.198 |
| Depressive symptoms | Model 2 | 0.016(-0.017,0.048) | 0.338 |
| Depressive symptoms | Model 3 | 0.015(-0.017,0.047) | 0.354 |
| Depressive symptoms | Model 4 | 0.013(-0.020,0.045) | 0.445 |

Abbreviations: BUN, blood urea nitrogen; CI, confidence interval.

Model 1 adjusted for no covariates.

Model 2 adjusted for age, gender, marital status, education, and residence.

Model 3 adjusted for age, gender, marital status, education, residence, alcohol, and smoking.

Model 4 adjusted for age, gender marital status, education, residence, alcohol, and smoking, BMI, hypertension, diabetes, and dyslipidemia.

**Table S4 Mediation analyses of depressive symptoms on BUNCr-induced cognition**

| **Model** | **Effect** | **Coefficient (95% *CI*)** | ***P*** |
| --- | --- | --- | --- |
| Model 1 | ACME | -0.0714(-0.1001,-0.0466) | <0.001 |
| Model 1 | ADE | -0.4449(-0.5876,-0.2982) | <0.001 |
| Model 1 | TE | -0.5163(-0.6619,-0.3679) | <0.001 |
| Model 1 | PM | 0.1382(0.0866,0.2132) | <0.001 |
| Model 2 | ACME | -0.0155(-0.0305,-0.0018) | 0.031 |
| Model 2 | ADE | -0.2010(-0.3359,-0.0698) | 0.004 |
| Model 2 | TE | -0.2165(-0.3532,-0.0857) | 0.003 |
| Model 2 | PM | 0.0714(0.0078,0.1993) | 0.034 |
| Model 3 | ACME | -0.0146(-0.0307,-0.0007) | 0.04 |
| Model 3 | ADE | -0.1962(-0.3327,-0.0595) | 0.006 |
| Model 3 | TE | -0.2108(-0.3475,-0.0745) | 0.002 |
| Model 3 | PM | 0.0692(0.0037,0.2295) | 0.042 |
| Model 4 | ACME | -0.0140(-0.0293,-0.0005) | 0.044 |
| Model 4 | ADE | -0.1852(-0.3186,-0.0502) | 0.009 |
| Model 4 | TE | -0.1992(-0.3331,-0.0638) | 0.005 |
| Model 4 | PM | 0.0703(0.0001,0.2459) | 0.049 |

Abbreviations: BUNCr, blood urea nitrogen to creatinine ratio; CI, confidence interval.

Model 1 adjusted for no covariates.

Model 2 adjusted for age, gender, marital status, education, and residence.

Model 3 adjusted for age, gender, marital status, education, residence, alcohol, and smoking.

Model 4 adjusted for age, gender marital status, education, residence, alcohol, and smoking, BMI, hypertension, diabetes, and dyslipidemia.

**Table S5 The relationships between BUNCr and cognitive function stratified by potential modifiers**

| **Modifiers** | **subgroup** | **Coefficient (95% *CI*)** | ***P*** | ***P_int_*** |
| --- | --- | --- | --- | --- |
| Age | <65 | -0.163(-0.318,-0.007) | 0.040 | 0.463 |
|  | >65 | -0.269(-0.514,-0.024) | 0.031 |  |
| Gender | femal | -0.275(-0.447,-0.102) | 0.002 | 0.141 |
|  | male | -0.072(-0.280,0.136) | 0.498 |  |
| Residence | urban | -0.095(-0.404,0.214) | 0.548 | 0.493 |
|  | rural | -0.213(-0.358,-0.067) | 0.004 |  |
| Alcohol | no | -0.215(-0.393,-0.036) | 0.018 | 0.712 |
|  | drink or quit | -0.166(-0.359,0.028) | 0.093 |  |
| Smoke | no | -0.309(-0.479,-0.139) | <0.001 | 0.031 |
|  | smoke or quit | -0.017(-0.225,0.191) | 0.874 |  |

Abbreviations: BUNCr, blood urea nitrogen to creatinine ratio; CI, confidence interval.

Model adjusted for age, gender marital status, education, residence, alcohol, and smoking, BMI, hypertension, diabetes, and dyslipidemia.

**Table S6 The relationships between BUNCr and depressive symptoms stratified by potential modifiers**

| **Modifiers** | **subgroup** | **Coefficient (95% *CI*)** | ***P*** | ***P_int_*** |
| --- | --- | --- | --- | --- |
| Age | <65 | 0.172(0.009,0.335) | 0.038 | 0.530 |
|  | >65 | 0.076(-0.181,0.334) | 0.562 |  |
| Gender | femal | 0.223(0.042,0.404) | 0.016 | 0.186 |
|  | male | 0.032(-0.187,0.251) | 0.776 |  |
| Residence | urban | -0.045(-0.370,0.280) | 0.786 | 0.203 |
|  | rural | 0.186(0.033,0.338) | 0.017 |  |
| Alcohol | no | 0.201(0.013,0.388) | 0.036 | 0.386 |
|  | drink or quit | 0.080(-0.123,0.284) | 0.440 |  |
| Smoke | no | 0.193(0.014,0.372) | 0.034 | 0.401 |
|  | smoke or quit | 0.074(-0.145,0.292) | 0.510 |  |

Abbreviations: BUNCr, blood urea nitrogen to creatinine ratio; CI, confidence interval.

Model adjusted for age, gender marital status, education, residence, alcohol, and smoking, BMI, hypertension, diabetes, and dyslipidemia.

**Table S7 The relationships between BUNCr and both cognitive function and depressive symptoms after multiple imputation**

| **Outcome** | **Model** | **Coefficient (95% *CI*)** | ***P*** |
| --- | --- | --- | --- |
| Cognition | Model 1 | -0.553(-0.680,-0.427) | <0.001 |
| Cognition | Model 2 | -0.366(-0.483,-0.249) | <0.001 |
| Cognition | Model 3 | -0.336(-0.454,-0.218) | <0.001 |
| Cognition | Model 4 | -0.315(-0.433,-0.197) | <0.001 |
| Depressive symptoms | Model 1 | 0.397(0.275,0.520) | <0.001 |
| Depressive symptoms | Model 2 | 0.333(0.211,0.455) | <0.001 |
| Depressive symptoms | Model 3 | 0.234(0.110,0.357) | <0.001 |
| Depressive symptoms | Model 4 | 0.218(0.095,0.342) | 0.001 |

Abbreviations: BUNCr, blood urea nitrogen to creatinine ratio; CI, confidence interval.

Model 1 adjusted for no covariates.

Model 2 adjusted for age, gender, marital status, education, and residence.

Model 3 adjusted for age, gender, marital status, education, residence, alcohol, and smoking.

Model 4 adjusted for age, gender marital status, education, residence, alcohol, and smoking, BMI, hypertension, diabetes, and dyslipidemia.

**Table S8 The relationships between BUNCr and both cognitive function and depressive symptoms after including participants under 45 years old**

| **Outcome** | **Model** | **Coefficient (95% *CI*)** | ***P*** |
| --- | --- | --- | --- |
| Cognition | Model 1 | -0.521(-0.664,-0.378) | <0.001 |
| Cognition | Model 2 | -0.227(-0.360,-0.095) | 0.001 |
| Cognition | Model 3 | -0.222(-0.355,-0.090) | 0.001 |
| Cognition | Model 4 | -0.204(-0.336,-0.072) | 0.003 |
| Depressive symptoms | Model 1 | 0.454(0.316,0.591) | <0.001 |
| Depressive symptoms | Model 2 | 0.163(0.023,0.302) | 0.022 |
| Depressive symptoms | Model 3 | 0.156(0.017,0.295) | 0.028 |
| Depressive symptoms | Model 4 | 0.147(0.008,0.286) | 0.038 |

Abbreviations: BUNCr, blood urea nitrogen to creatinine ratio; CI, confidence interval.

Model 1 adjusted for no covariates.

Model 2 adjusted for age, gender, marital status, education, and residence.

Model 3 adjusted for age, gender, marital status, education, residence, alcohol, and smoking.

Model 4 adjusted for age, gender marital status, education, residence, alcohol, and smoking, BMI, hypertension, diabetes, and dyslipidemia.

**Table S9 The relationships between BUNCr and both cognitive function and depressive symptoms after including participants with extreme BMI**

| **Outcome** | **Model** | **Coefficient (95% *CI*)** | ***P*** |
| --- | --- | --- | --- |
| Cognition | Model 1 | -0.523(-0.667,-0.380) | <0.001 |
| Cognition | Model 2 | -0.221(-0.354,-0.088) | 0.001 |
| Cognition | Model 3 | -0.216(-0.349,-0.083) | 0.002 |
| Cognition | Model 4 | -0.205(-0.338,-0.072) | 0.003 |
| Depressive symptoms | Model 1 | 0.460(0.322,0.598) | <0.001 |
| Depressive symptoms | Model 2 | 0.154(0.014,0.294) | 0.031 |
| Depressive symptoms | Model 3 | 0.147(0.007,0.287) | 0.039 |
| Depressive symptoms | Model 4 | 0.142(0.002,0.282) | 0.047 |

Abbreviations: BUNCr, blood urea nitrogen to creatinine ratio; BMI, body mass index; CI, confidence interval.

Model 1 adjusted for no covariates.

Model 2 adjusted for age, gender, marital status, education, and residence.

Model 3 adjusted for age, gender, marital status, education, residence, alcohol, and smoking.

Model 4 adjusted for age, gender marital status, education, residence, alcohol, and smoking, BMI, hypertension, diabetes, and dyslipidemia.

**Table S10 The relationships between BUNCr and both episodic memory and mental status**

| **Outcome** | **Model** | **Coefficient (95% *CI*)** | ***P*** |
| --- | --- | --- | --- |
| Episodic memory | Model 1 | -0.359(-0.466,-0.252) | <0.001 |
| Episodic memory | Model 2 | -0.160(-0.261,-0.059) | 0.002 |
| Episodic memory | Model 3 | -0.155(-0.256,-0.054) | 0.003 |
| Episodic memory | Model 4 | -0.141(-0.242,-0.040) | 0.006 |
| Mental status | Model 1 | -0.157(-0.206,-0.109) | <0.001 |
| Mental status | Model 2 | -0.057(-0.103,-0.011) | 0.015 |
| Mental status | Model 3 | -0.056(-0.102,-0.010) | 0.018 |
| Mental status | Model 4 | -0.052(-0.098,-0.006) | 0.027 |

Abbreviations: BUNCr, blood urea nitrogen to creatinine ratio; CI, confidence interval.

Model 1 adjusted for no covariates.

Model 2 adjusted for age, gender, marital status, education, and residence.

Model 3 adjusted for age, gender, marital status, education, residence, alcohol, and smoking.

Model 4 adjusted for age, gender marital status, education, residence, alcohol, and smoking, BMI, hypertension, diabetes, and dyslipidemia.

**Table S11 Mediation analyses of depressive symptoms on BUNCr-induced cognition after multiple imputation**

| **Model** | **effect** | **Coefficient (95% *CI*)** | ***P*** |
| --- | --- | --- | --- |
| Model 1 | ACME | -0.065(-0.0899,-0.0417) | <0.001 |
| Model 1 | ADE | -0.4882(-0.6103,-0.362) | <0.001 |
| Model 1 | TE | -0.5532(-0.6756,-0.4256) | <0.001 |
| Model 1 | PM | 0.1174(0.0749,0.1723) | <0.001 |
| Model 2 | ACME | -0.0122(-0.0276,0.0027) | 0.103 |
| Model 2 | ADE | -0.2677(-0.3902,-0.1512) | <0.001 |
| Model 2 | TE | -0.2799(-0.4025,-0.1621) | <0.001 |
| Model 2 | PM | 0.0437(-0.0112,0.1111) | 0.103 |
| Model 3 | ACME | -0.0116(-0.0266,0.0027) | 0.104 |
| Model 3 | ADE | -0.2643(-0.3841,-0.1496) | <0.001 |
| Model 3 | TE | -0.2759(-0.3984,-0.1596) | <0.001 |
| Model 3 | PM | 0.0420(-0.0109,0.111) | 0.104 |
| Model 4 | ACME | -0.0095(-0.0238,0.0044) | 0.180 |
| Model 4 | ADE | -0.2416(-0.3542,-0.1185) | <0.001 |
| Model 4 | TE | -0.2512(-0.3642,-0.1259) | <0.001 |
| Model 4 | PM | 0.0379(-0.0188,0.1135) | 0.180 |

Abbreviations: BUNCr, blood urea nitrogen to creatinine ratio; CI, confidence interval.

Model 1 adjusted for no covariates.

Model 2 adjusted for age, gender, marital status, education, and residence.

Model 3 adjusted for age, gender, marital status, education, residence, alcohol, and smoking.

Model 4 adjusted for age, gender marital status, education, residence, alcohol, and smoking, BMI, hypertension, diabetes, and dyslipidemia.

**Table S12 Mediation analyses of depressive symptoms on BUNCr-induced cognition after including participants under 45 years old**

| **Model** | **Effect** | **Coefficient (95% *CI*)** | ***P*** |
| --- | --- | --- | --- |
| Model 1 | ACME | -0.0687(-0.0952,-0.0449) | <0.001 |
| Model 1 | ADE | -0.4522(-0.5915,-0.3118) | <0.001 |
| Model 1 | TE | -0.5209(-0.6613,-0.3762) | <0.001 |
| Model 1 | PM | 0.1318(0.0839,0.2031) | <0.001 |
| Model 2 | ACME | -0.0153(-0.0315,-0.0017) | 0.031 |
| Model 2 | ADE | -0.2119(-0.3445,-0.0698) | 0.003 |
| Model 2 | TE | -0.2272(-0.3637,-0.0859) | 0.001 |
| Model 2 | PM | 0.0673(0.0059,0.1894) | 0.032 |
| Model 3 | ACME | -0.0145(-0.0299,-0.0012) | 0.033 |
| Model 3 | ADE | -0.2077(-0.3445,-0.0718) | 0.003 |
| Model 3 | TE | -0.2222(-0.3592,-0.0837) | 0.002 |
| Model 3 | PM | 0.0651(0.0053,0.1876) | 0.035 |
| Model 4 | ACME | -0.0134(-0.0286,-0.0010) | 0.035 |
| Model 4 | ADE | -0.1907(-0.3358,-0.0551) | 0.004 |
| Model 4 | TE | -0.2041(-0.3497,-0.0667) | 0.001 |
| Model 4 | PM | 0.0655(0.0040,0.2299) | 0.036 |

Abbreviations: BUNCr, blood urea nitrogen to creatinine ratio; CI, confidence interval.

Model 1 adjusted for no covariates.

Model 2 adjusted for age, gender, marital status, education, and residence.

Model 3 adjusted for age, gender, marital status, education, residence, alcohol, and smoking.

Model 4 adjusted for age, gender marital status, education, residence, alcohol, and smoking, BMI, hypertension, diabetes, and dyslipidemia.

**Table S13 Mediation analyses of depressive symptoms on BUNCr-induced cognition after including participants with extreme BMI**

| **Model** | **Effect** | **Coefficient (95% *CI*)** | ***P*** |
| --- | --- | --- | --- |
| Model 1 | ACME | -0.0717(-0.1005,-0.0466) | <0.001 |
| Model 1 | ADE | -0.4517(-0.5982,-0.3074) | <0.001 |
| Model 1 | TE | -0.5234(-0.6697,-0.3801) | <0.001 |
| Model 1 | PM | 0.137(0.083,0.2074) | <0.001 |
| Model 2 | ACME | -0.0151(-0.0303,-0.0007) | 0.037 |
| Model 2 | ADE | -0.2058(-0.3384,-0.0782) | 0.002 |
| Model 2 | TE | -0.2209(-0.3550,-0.0911) | 0.001 |
| Model 2 | PM | 0.0685(0.003,0.1924) | 0.038 |
| Model 3 | ACME | -0.0142(-0.0301,-0.0001) | 0.05 |
| Model 3 | ADE | -0.2014(-0.3312,-0.0662) | 0.005 |
| Model 3 | TE | -0.2156(-0.3472,-0.0796) | 0.002 |
| Model 3 | PM | 0.0660(-0.0007,0.2238) | 0.052 |
| Model 4 | ACME | -0.0137(-0.0296,-0.0002) | 0.047 |
| Model 4 | ADE | -0.1910(-0.3213,-0.0551) | 0.006 |
| Model 4 | TE | -0.2047(-0.3360,-0.0693) | 0.003 |
| Model 4 | PM | 0.0668(0.0002,0.2273) | 0.050 |

Abbreviations: BUNCr, blood urea nitrogen to creatinine ratio; CI, confidence interval.

Model 1 adjusted for no covariates.

Model 2 adjusted for age, gender, marital status, education, and residence.

Model 3 adjusted for age, gender, marital status, education, residence, alcohol, and smoking.

Model 4 adjusted for age, gender marital status, education, residence, alcohol, and smoking, BMI, hypertension, diabetes, and dyslipidemia.

**Table S14 Mediation analyses of depressive symptoms on the relationship between BUNCr and episodic memory**

| **Model** | **Effect** | **Coefficient (95% *CI*)** | ***P*** |
| --- | --- | --- | --- |
| Model 1 | ACME | -0.0478(-0.0663,-0.0312) | <0.001 |
| Model 1 | ADE | -0.3112(-0.4213,-0.2072) | <0.001 |
| Model 1 | TE | -0.3590(-0.4692,-0.2539) | <0.001 |
| Model 1 | PM | 0.1331(0.0812,0.2047) | <0.001 |
| Model 2 | ACME | -0.0104(-0.0204,-0.0002) | 0.045 |
| Model 2 | ADE | -0.1493(-0.2515,-0.0519) | 0.002 |
| Model 2 | TE | -0.1597(-0.2621,-0.0612) | 0.002 |
| Model 2 | PM | 0.0650(0.0009,0.1969) | 0.047 |
| Model 3 | ACME | -0.0098(-0.021,-0.0004) | 0.039 |
| Model 3 | ADE | -0.1454(-0.2477,-0.0444) | 0.004 |
| Model 3 | TE | -0.1551(-0.2581,-0.0531) | 0.004 |
| Model 3 | PM | 0.0629(0.0008,0.2064) | 0.043 |
| Model 4 | ACME | -0.0090(-0.0193,0.0001) | 0.055 |
| Model 4 | ADE | -0.1317(-0.2329,-0.026) | 0.017 |
| Model 4 | TE | -0.1407(-0.2411,-0.0355) | 0.013 |
| Model 4 | PM | 0.0637(-0.0034,0.2422) | 0.068 |

Abbreviations: BUNCr, blood urea nitrogen to creatinine ratio; CI, confidence interval.

Model 1 adjusted for no covariates.

Model 2 adjusted for age, gender, marital status, education, and residence.

Model 3 adjusted for age, gender, marital status, education, residence, alcohol, and smoking.

Model 4 adjusted for age, gender marital status, education, residence, alcohol, and smoking, BMI, hypertension, diabetes, and dyslipidemia.

**Table S15 Mediation analyses of depressive symptoms on the relationship between BUNCr and mental status**

| **Model** | **Effect** | **Coefficient (95% *CI*)** | ***P*** |
| --- | --- | --- | --- |
| Model 1 | ACME | -0.0236(-0.0327,-0.0151) | <0.001 |
| Model 1 | ADE | -0.1337(-0.1875,-0.0826) | <0.001 |
| Model 1 | TE | -0.1573(-0.2116,-0.1065) | <0.001 |
| Model 1 | PM | 0.1500(0.0916,0.2421) | <0.001 |
| Model 2 | ACME | -0.0051(-0.0103,-0.0006) | 0.033 |
| Model 2 | ADE | -0.0517(-0.0997,-0.0048) | 0.026 |
| Model 2 | TE | -0.0568(-0.1058,-0.0109) | 0.01 |
| Model 2 | PM | 0.0896(0.0048,0.4139) | 0.043 |
| Model 3 | ACME | -0.0048(-0.0101,-0.0001) | 0.047 |
| Model 3 | ADE | -0.0508(-0.0952,-0.0019) | 0.038 |
| Model 3 | TE | -0.0557(-0.1003,-0.0057) | 0.021 |
| Model 3 | PM | 0.0868(-0.009,0.5071) | 0.068 |
| Model 4 | ACME | -0.0046(-0.0098,-0.0001) | 0.048 |
| Model 4 | ADE | -0.0471(-0.0928,-0.0011) | 0.042 |
| Model 4 | TE | -0.0517(-0.0967,-0.0066) | 0.029 |
| Model 4 | PM | 0.0885(-0.0149,0.4949) | 0.073 |

Abbreviations: BUNCr, blood urea nitrogen to creatinine ratio; CI, confidence interval.

Model 1 adjusted for no covariates.

Model 2 adjusted for age, gender, marital status, education, and residence.

Model 3 adjusted for age, gender, marital status, education, residence, alcohol, and smoking.

Model 4 adjusted for age, gender marital status, education, residence, alcohol, and smoking, BMI, hypertension, diabetes, and dyslipidemia.

**Table S16 The relationships between BUNCr and cognitive function stratified by potential modifiers after multiple imputation**

| **Modifiers** | **subgroup** | **Coefficient (95% *CI*)** | ***P*** | ***P_int_*** |
| --- | --- | --- | --- | --- |
| Age | <65 | -0.22(-0.358,-0.083) | 0.002 | 0.375 |
|  | >65 | -0.339(-0.568,-0.111) | 0.004 |  |
| Gender | femal | -0.347(-0.502,-0.193) | <0.001 | 0.054 |
|  | male | -0.108(-0.297,0.080) | 0.26 |  |
| Residence | urban | -0.231(-0.512,0.050) | 0.106 | 0.878 |
|  | rural | -0.255(-0.386,-0.125) | <0.001 |  |
| Alcohol | no | -0.311(-0.471,-0.152) | <0.001 | 0.262 |
|  | drink or quit | -0.178(-0.353,-0.003) | 0.047 |  |
| Smoke | no | -0.400(-0.551,-0.248) | <0.001 | 0.002 |
|  | smoke or quit | -0.019(-0.208,0.171) | 0.847 |  |

Abbreviations: BUNCr, blood urea nitrogen to creatinine ratio; CI, confidence interval.

Model adjusted for age, gender marital status, education, residence, alcohol, and smoking, BMI, hypertension, diabetes, and dyslipidemia.

**Table S17 The relationships between BUNCr and depressive symptoms stratified by potential modifiers after multiple imputation**

| **Modifiers** | **subgroup** | **Coefficient (95% *CI*)** | ***P*** | ***P_int_*** |
| --- | --- | --- | --- | --- |
| Age | <65 | 0.109(-0.034,0.252) | 0.135 | 0.562 |
|  | >65 | 0.028(-0.209,0.265) | 0.815 |  |
| Gender | femal | 0.129(-0.032,0.289) | 0.115 | 0.431 |
|  | male | 0.028(-0.168,0.223) | 0.783 |  |
| Residence | urban | -0.065(-0.357,0.226) | 0.660 | 0.254 |
|  | rural | 0.119(-0.016,0.255) | 0.084 |  |
| Alcohol | no | 0.112(-0.054,0.277) | 0.187 | 0.674 |
|  | drink or quit | 0.060(-0.122,0.241) | 0.521 |  |
| Smoke | no | 0.115(-0.043,0.272) | 0.155 | 0.594 |
|  | smoke or quit | 0.047(-0.150,0.243) | 0.642 |  |

Abbreviations: BUNCr, blood urea nitrogen to creatinine ratio; CI, confidence interval.

Model adjusted for age, gender marital status, education, residence, alcohol, and smoking, BMI, hypertension, diabetes, and dyslipidemia.

**Table S18 The relationships between BUNCr and cognitive function stratified by potential modifiers after including participants with extreme BMI**

| **Modifiers** | **subgroup** | **Coefficient (95% *CI*)** | ***P*** | ***P_int_*** |
| --- | --- | --- | --- | --- |
| Age | <65 | -0.178(-0.331,-0.024) | 0.024 | 0.508 |
|  | >65 | -0.274(-0.518,-0.029) | 0.028 |  |
| Gender | femal | -0.286(-0.457,-0.115) | 0.001 | 0.138 |
|  | male | -0.083(-0.291,0.125) | 0.434 |  |
| Residence | urban | -0.113(-0.421,0.194) | 0.470 | 0.522 |
|  | rural | -0.223(-0.368,-0.079) | 0.003 |  |
| Alcohol | no | -0.229(-0.407,-0.052) | 0.011 | 0.675 |
|  | drink or quit | -0.174(-0.367,0.019) | 0.076 |  |
| Smoke | no | -0.317(-0.486,-0.148) | <0.001 | 0.036 |
|  | smoke or quit | -0.034(-0.241,0.173) | 0.749 |  |

Abbreviations: BUNCr, blood urea nitrogen to creatinine ratio; BMI, body mass index; CI, confidence interval.

Model adjusted for age, gender marital status, education, residence, alcohol, and smoking, BMI, hypertension, diabetes, and dyslipidemia.

**Table S19 The relationships between BUNCr and depressive symptoms stratified by potential modifiers after including participants under 45 years old**

| **Modifiers** | **subgroup** | **Coefficient (95% *CI*)** | ***P*** | ***P_int_*** |
| --- | --- | --- | --- | --- |
| Age | <65 | 0.173(0.011,0.334) | 0.037 | 0.545 |
|  | >65 | 0.080(-0.177,0.337) | 0.541 |  |
| Gender | femal | 0.231(0.051,0.410) | 0.012 | 0.149 |
|  | male | 0.023(-0.195,0.241) | 0.834 |  |
| Residence | urban | -0.047(-0.370,0.276) | 0.776 | 0.193 |
|  | rural | 0.188(0.036,0.340) | 0.015 |  |
| Alcohol | no | 0.207(0.021,0.393) | 0.029 | 0.343 |
|  | drink or quit | 0.076(-0.126,0.278) | 0.462 |  |
| Smoke | no | 0.201(0.023,0.378) | 0.027 | 0.342 |
|  | smoke or quit | 0.066(-0.152,0.283) | 0.553 |  |

Abbreviations: BUNCr, blood urea nitrogen to creatinine ratio; CI, confidence interval.

Model adjusted for age, gender marital status, education, residence, alcohol, and smoking, BMI, hypertension, diabetes, and dyslipidemia.

**Table S20 The relationships between BUNCr and cognitive function stratified by potential modifiers after including participants with extreme BMI**

| **Modifiers** | **subgroup** | **Coefficient (95% *CI*)** | ***P*** | ***P_int_*** |
| --- | --- | --- | --- | --- |
| Age | <65 | -0.184(-0.339,-0.029) | 0.020 | 0.612 |
|  | >65 | -0.258(-0.503,-0.013) | 0.039 |  |
| Gender | femal | -0.279(-0.451,-0.106) | 0.002 | 0.185 |
|  | male | -0.096(-0.305,0.112) | 0.365 |  |
| Residence | urban | -0.101(-0.420,0.20) | 0.486 | 0.508 |
|  | rural | -0.224(-0.370,-0.079) | 0.002 |  |
| Alcohol | no | -0.224(-0.402,-0.045) | 0.014 | 0.755 |
|  | drink or quit | -0.182(-0.376,0.011) | 0.065 |  |
| Smoke | no | -0.313(-0.483,-0.143) | <0.001 | 0.046 |
|  | smoke or quit | -0.042(-0.250,0.166) | 0.693 |  |

Abbreviations: BUNCr, blood urea nitrogen to creatinine ratio; BMI, body mass index; CI, confidence interval.

Model adjusted for age, gender marital status, education, residence, alcohol, and smoking, BMI, hypertension, diabetes, and dyslipidemia.

**Table S21 The relationships between BUNCr and depressive symptoms stratified by potential modifiers after including participants with extreme BMI**

| **Modifiers** | **subgroup** | **Coefficient (95% *CI*)** | ***P*** | ***P_int_*** |
| --- | --- | --- | --- | --- |
| Age | <65 | 0.171(0.008,0.334) | 0.040 | 0.497 |
|  | >65 | 0.067(-0.190,0.324) | 0.609 |  |
| Gender | femal | 0.212(0.032,0.393) | 0.021 | 0.228 |
|  | male | 0.038(-0.180,0.257) | 0.731 |  |
| Residence | urban | -0.042(-0.367,0.283) | 0.801 | 0.22 |
|  | rural | 0.180(0.028,0.333) | 0.021 |  |
| Alcohol | no | 0.188(0.001,0.376) | 0.049 | 0.466 |
|  | drink or quit | 0.087(-0.116,0.290) | 0.401 |  |
| Smoke | no | 0.183(0.005,0.362) | 0.045 | 0.469 |
|  | smoke or quit | 0.080(-0.139,0.298) | 0.474 |  |

Abbreviations: BUNCr, blood urea nitrogen to creatinine ratio; BMI, body mass index; CI, confidence interval.

Model adjusted for age, gender marital status, education, residence, alcohol, and smoking, BMI, hypertension, diabetes, and dyslipidemia.

**Table S22 The relationships between BUNCr and episodic memory stratified by potential modifiers**

| **Modifiers** | **subgroup** | **Coefficient (95% *CI*)** | ***P*** | ***P_int_*** |
| --- | --- | --- | --- | --- |
| Age | <65 | -0.129(-0.247,-0.012) | 0.031 | 0.709 |
|  | >65 | -0.17(-0.356,0.015) | 0.072 |  |
| Gender | femal | -0.201(-0.331,-0.07) | 0.003 | 0.158 |
|  | male | -0.053(-0.211,0.105) | 0.511 |  |
| Residence | urban | -0.09(-0.324,0.145) | 0.453 | 0.637 |
|  | rural | -0.151(-0.262,-0.041) | 0.007 |  |
| Alcohol | no | -0.165(-0.300,-0.029) | 0.017 | 0.601 |
|  | drink or quit | -0.112(-0.259,0.035) | 0.134 |  |
| Smoke | no | -0.231(-0.360,-0.102) | <0.001 | 0.028 |
|  | smoke or quit | -0.005(-0.163,0.153) | 0.951 |  |

Abbreviations: BUNCr, blood urea nitrogen to creatinine ratio; CI, confidence interval.

Model adjusted for age, gender marital status, education, residence, alcohol, and smoking, BMI, hypertension, diabetes, and dyslipidemia.

**Table S23 The relationships between BUNCr and mental status stratified by potential modifiers**

| **Modifiers** | **subgroup** | **Coefficient (95% *CI*)** | ***P*** | ***P_int_*** |
| --- | --- | --- | --- | --- |
| Age | <65 | -0.033(-0.087,0.020) | 0.222 | 0.191 |
|  | >65 | -0.099(-0.183,-0.015) | 0.022 |  |
| Gender | femal | -0.074(-0.133,-0.015) | 0.015 | 0.247 |
|  | male | -0.019(-0.091,0.053) | 0.604 |  |
| Residence | urban | -0.005(-0.112,0.102) | 0.927 | 0.342 |
|  | rural | -0.062(-0.112,-0.011) | 0.016 |  |
| Alcohol | no | -0.05(-0.112,0.012) | 0.111 | 0.937 |
|  | drink or quit | -0.054(-0.120,0.013) | 0.116 |  |
| Smoke | no | -0.078(-0.137,-0.019) | 0.009 | 0.156 |
|  | smoke or quit | -0.012(-0.084,0.060) | 0.746 |  |

Abbreviations: BUNCr, blood urea nitrogen to creatinine ratio; CI, confidence interval.

Model adjusted for age, gender marital status, education, residence, alcohol, and smoking, BMI, hypertension, diabetes, and dyslipidemia.
